# Assessing liquid light guides in diffuse correlation spectroscopy systems

**DOI:** 10.1364/BOE.571835

**Published:** 2025-11-04

**Authors:** Yuanzhe Zhang, Mingliang Pan, Chenxu Li, Ziao Jiao, Yuanyuan Hua, Ahmet T. Erdogan, Robert K. Henderson, David Day-Uei Li

**Affiliations:** 1 University of Strathclyde, Department of Biomedical Engineering, Glasgow, UK; 2 University of Edinburgh, Institute for Micro and Nano Systems (IMNS), School of Engineering, Edinburgh, UK

## Abstract

Diffuse correlation spectroscopy (DCS) is a widely used noninvasive optical technique for measuring tissue blood flow. Accurate blood flow estimation with DCS requires a high signal-to-noise ratio (SNR), but achieving high SNR is often limited by safety constraints on the optical irradiance (maximum permissible exposure) that can be delivered to tissue. To overcome this limitation, we investigated the possibility of replacing the conventional multi-mode fiber (MMF) with a liquid light guide (LLG) for illumination. The LLG provides a more uniform illumination profile and higher photon throughput to the tissue under the same irradiance limit, resulting in a significantly increased detected photon count rate and enhanced SNR. In experiments under identical power-density conditions, the LLG-based system achieved approximately a three-fold increase in SNR compared to the traditional MMF configuration. This improvement arises from the uniform beam profile and efficient light delivery of the LLG, which permits safe use of higher total power. These results indicate that LLG illumination effectively enhances DCS sensitivity without exceeding safety limits, potentially enabling more sensitive and accurate blood flow monitoring in biomedical applications.

## 1. Introduction

Diffuse correlation spectroscopy (DCS) is a non-invasive near-infrared optical method for deep-tissue blood flow (BF) measurement. In biological tissues, BF delivers oxygen and nutrients and clears metabolic waste, making it a fundamental indicator of tissue perfusion and health [1]. DCS quantifies BF by measuring fluctuations in the scattered light intensity: the normalized intensity autocorrelation function (ACF, $g_{2}$ ) is related via the Siegert relation to the field autocorrelation $g_{1}$ :

(1) $$g_{2}(\tau )=1+\beta {\left|g_{1}(\tau )\right|}^{2},$$

where β depends on the laser stability, the coherence length, and the number of speckles detected [2]. The measured $g_{2}$ is calculated as:

(2) $$g_{2}(\tau )=\frac{\left\langle I(t)I(t+\tau )\right\rangle }{\langle I\left(t\right)\rangle^{2}},$$

where ⟨…⟩ denotes the average over the integration time, and I(t) is the measured light intensity series. The decay of $g_{1}$ is driven by moving red blood cells (RBCs) in microvasculature, so that DCS is highly sensitive to local blood dynamics. In practice, DCS typically uses single-mode fibers and single-photon detectors to collect photons and compute *g_2_*(*τ*) from their temporal fluctuations.

Due to its real-time readout, safety, and portability, DCS has been applied widely in physiological and clinical studies. For example, DCS has enabled continuous bedside monitoring of cerebral hemodynamics (e.g., neurovascular coupling and brain activation), cardiac and cerebrovascular function (such as stroke and neonatal brain perfusion), and tumor vascularization for diagnosis and therapy assessment [3]. In all these cases, the blood flow index (BFi) derived from the $g_{2}(\tau )$ curves provide a biomarker of tissue perfusion and metabolic state.

Despite these advantages, extracting an accurate BFi in tissue is challenging. Photon shot noise and detector noise can significantly distort the measured $g_{2}(\tau )$ , degrading its signal-to-noise ratio (SNR) and biasing the flow index. In DCS, single-photon detectors record photon arrival times and the correlator accumulates $g_{2}(\tau )$ , however, when extremely low photon count rate detected, photon statistical noise cannot be overlooked. In fact, the variance of $g_{2}(\tau )$ is dominated by photon counting statistics. Any excess noise (e.g. detector dark counts, ambient light) further reduces SNR and thus affects the precision of BFi.

To understand and mitigate this noise, theoretical models have been developed. The classic noise model of Koppel (1974) for fluorescence correlation spectroscopy was adapted for DCS by Zhou *et al* [4]. This model yields an analytical expression for the standard deviation σ(τ) of the measured correlation. In the common regime of slow decorrelation and low count rates, the model is simplified to [5, pp. 37–41]:

(3) $$\sigma (\tau )\approx \frac{1}{I}\sqrt{\frac{1}{t}}\sqrt{\frac{1+\beta e^{-\Gamma \tau }}{T}},$$

where *I* is the detected photon count rate, *t* is the total measurement time, *T* is the correlator bin width, β is the coherence factor, and Γ is the decay rate related to flow dynamics. Equation (3) shows explicitly that the noise amplitude is inversely proportional to *I*: doubling the count rate (for fixed *t* and *T*) halves the standard deviation. This noise model provides a quantitative framework for optimizing DCS parameters and assessing the uncertainty in flow measurements.

A typical DCS setup comprises a near-infrared laser with a long coherence length, a multi-mode fiber (MMF) for light delivery, single-mode fibers (SMF) for detection, single-photon detectors, and a correlator. However, the laser must adhere to the maximum permissible exposure (MPE) limits. For instance, ANSI Z136.1 specifies that a 785 nm continuous-wave laser has an MPE of 0.28 W/cm^2^ for prolonged skin exposure. This power limitation constrains the DCS’s ability to achieve high temporal resolution and to utilize significant source–to-detector separations (*ρ*), thereby limiting the ability to probe deeper blood flow signals [3]. Researchers have attempted to expand the distance between fiber tips and the tissue surface or to use a lens to expand the beam spot in order to reduce the skin’s irradiance [6,7].

The output from a single-mode laser coupled into an MMF typically maintains a Gaussian-like intensity distribution [8]. A substantial portion of the optical power remains concentrated near the central axis. Safety regulations, such as those specified in ANSI Z136.1 and IEC 60825, define exposure limits based on power averaged over a fixed, small-area aperture. As a result, geometric beam expansion yields only modest increases in total allowable optical power. In contrast, a beam with a more uniform (top-hat) intensity profile distributes optical energy more evenly, thereby reducing localized exposure and potentially allowing for higher permissible power without exceeding safety thresholds. A comparative analysis of Gaussian and uniform beam profiles, and their implications for exposure safety, is discussed further in the Discussion section. Additionally, reflected light from tissue surfaces can pose ocular safety risks and reduce photon delivery to the target.

To address these challenges, we implemented a liquid light guide (LLG) as the illumination conduit in our system, which produced a visibly more uniform output profile than traditional MMF-based delivery. LLGs are constructed with a transparent liquid core enclosed in a flexible, typically Teflon- or polymer-based sheath. Light propagates through the core via total internal reflection, eliminating the packing inefficiencies associated with fiber bundles and yielding higher overall transmission efficiency. Compared to MMFs, LLGs offer significantly larger core diameters (typically 0.2–10 mm) while maintaining excellent flexibility, making them well-suited for optical delivery in systems with complex geometries [9]. LLGs have been employed in various optical applications including laser surgery [10], fluorescence imaging [11], and radiation detection [12]. Notably, James *et al* [13]. utilized an LLG in a heterodyne parallel speckle detection system for photon collection.

In this study, we aim to compare LLG and MMF configurations in DCS through phantom experiments, evaluate beam uniformity improvements, quantify SNR enhancement, and validate the feasibility via in vivo testing. In short, these investigations demonstrate the viability and advantages of incorporating LLG-based illumination into conventional DCS systems, particularly by enabling greater light input within system limits, resulting in improved SNR.

## 2. Methods

### 2.1. Experimental setup

We conducted two experiments to compare the performance consistency between MMF and LLG configurations in DCS. The continuous-wave (CW) DCS system employed a DL785-100-S laser (785 nm, > 10 m coherence length, 100 mW maximum output) that produced a near-TEM_00_ circular beam with a beam quality factor (M^2^) of approximately 1.1–1.3. Laser output power was attenuated using neutral density filters to achieve the desired illumination level. The beam was then coupled into either the MMF or LLG using a short focal length aspheric lens (F110FC-780). The output power from the delivery fiber was continuously monitored with a calibrated power meter.

Two illumination pathways were evaluated: (1) a conventional MMF (600 µm core diameter, Numerical Aperture (NA) = 0.22, Thorlabs M160L01), and (2) an LLG (model LLG5-4Z, 5 mm core diameter), which provides over 80% transmission efficiency at 785 nm. For both configurations, photon detection was performed using a single-photon avalanche diode (SPAD; ID101-SMF20, Swiss Quantum), and the photon arrival data were processed by a hardware time tagger (SPC-QC-104, Becker & Hickl GmbH) to compute the intensity autocorrelation function, $g_{2}(\tau )$ .

### 2.2. Phantom experiments for consistency validation

**Fig. 1.**
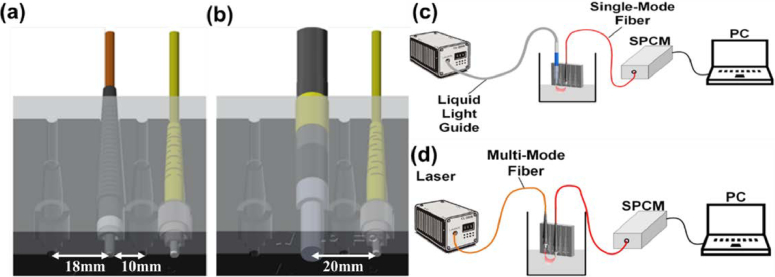
Experimental setup and probe design for comparing MMF and LLG illumination in DCS. (a) and (b) Mounting models for MMF and LLG illumination, each fixed in a customized 3D-printed holder designed to maintain the same source–detector geometry and to minimize stray reflections between the liquid surface and the environment. (c) and (d) Schematics of the experimental configuration showing the laser, delivery fiber (MMF or LLG).

The first experimental configuration employed a conventional MMF, whereas the second utilized an LLG (LLG5-4Z). Both setups used the same phantom, consisting of semi-skimmed milk diluted 1:3 with water. A 3D-printed positioning fixture (Fig. 1) was used to maintain consistent alignment and geometry between the illumination and detection fibers across all measurements. Experiments were performed at six source–detector separations (*ρ* = 10, 12, 14, 16, 18, 20). At *ρ* = 20 mm, two different laser output powers (∼35 mW and ∼90 mW) were also tested. For each condition, $g_{2}(\tau )$ was recorded, and the Brownian diffusion coefficient $D_{B}$ was extracted by fitting the decay of $g_{2}(\tau )$ using the semi-infinite homogeneous analytical solution. In this model, motion can be described as the random Brownian diffusion of scatterers, as illustrated by the Einstein–Stokes relation as:

(4) $$D_{B}=\frac{k_{B}T}{6\pi \eta r},$$

where $k_{B}$ is Boltzmann’s constant, T is the absolute temperature, η is the dynamic viscosity of the medium, and *r* is the effective particle radius.

A theoretical model based on the correlation diffusion equation (CDE) was applied to extract quantitative BFi from the measured $g_{2}(\tau )$ . The Green’s function solution to the CDE can be expressed in Eq. (5):

(5) $$G_{1}(\rho ,\tau )=\frac{3\mu_{s}^{\prime }}{4\pi }\left[\frac{e^{\left(-Kr_{1}\right)}}{r_{1}}-\frac{e^{\left(-Kr_{2}\right)}}{r_{2}}\right],$$

where $K^{2}=3\mu_{a}\mu_{s}^{\prime }+\mu_{s}^{\prime^{2}}k_{0}^{2}\alpha \left\langle \Delta r^{2}(\tau )\right\rangle$ , $k_{0}$ is the wavenumber of light in the medium, $r_{1}=\sqrt{\rho^{2}+z_{0}^{2}}$ , $r_{2}=\sqrt{\rho^{2}+{\left(z_{0}+2z_{b}\right)}^{2}}$ , $z_{0}=1/(\mu_{a}+\mu_{s}^{\prime })$ , $z_{b}=2(1+R_{\text{eff}})/(3\mu_{s}^{\prime }(1-R_{\text{eff}})),$ and $R_{eff}=-1.44n^{-2}+0.71n^{-1}+0.668+0.064n$ is the effective reflection coefficient. Also $n=n_{0}/n_{air}$ , where $n_{0}$ is the medium refractive index and $n_{air}$ is the air refractive index. For the milk phantom, the absorption coefficient ( $\mu_{a}$ ) was set to 0.0025 mm^−1^ and the reduced scattering coefficient ( $\mu_{s}^{\prime }$ ) was set to 1.0 mm^−1^. In diffusive media, $\left\langle \Delta r^{2}(\tau )\right\rangle =6D_{B}\tau$ . The product $\alpha D_{B}$ is defined as BFi [14,15]. By fitting Eq. (2), the BFi ( $\alpha D_{B}$ ) can be extracted, where α is defined as the fraction of moving scatterers relative to the total scatterer population, typically assumed to be 1 in homogeneous phantoms [16]. Additional temperature-controlled phantom tests were performed by heating the diluted milk solution in a water bath to 25, 30, and 35 °C, and recording $g_{2}(\tau )$ under both MMF and LLG illumination.

### 2.3. Beam profile analysis

We evaluated the beam profiles of the LLG and MMF configurations using a CMOS camera. To accommodate the larger core diameter of the LLG, the camera sensor was positioned approximately 1 mm from the LLG tip to capture the full beam spot. For MMFs with core diameters of 200 µm (NA = 0.39), 600 µm (NA = 0.22), and 1000 µm (NA = 0.50), the corresponding fiber-to-camera distances were calculated to produce a comparable beam diameter of approximately 5 mm. These distances were determined using the relation:

(6) $$L=\frac{D}{2\tan(\arcsin(\text{NA}))},$$

where *L* is the required fiber-to-camera distance, *D* is the desired beam diameter (matching that of the LLG), and NA is the fiber’s numerical aperture.

To prevent pixel saturation from high-intensity speckles, 200 consecutive frames were acquired using the camera’s minimum exposure time (22 µs) and lowest gain setting. The laser output was attenuated from 100 mW to ∼1 mW using a neutral density filter. The captured frames were then averaged, and background noise was subtracted to generate the representative beam profile used in subsequent analysis.

### 2.4. SNR analysis

To characterize DCS measurement noise and SNR, we recorded 100 consecutive $g_{2}$ curves on the milk phantom at *ρ* = 20 mm for both the LLG and MMF configurations. The integration time was 1 s for each measurement, and the correlator employed a multi-tau binning strategy to compute the autocorrelation. For each delay time τ, we calculated the standard deviation of $g_{2}(\tau )$ across the 100 measurements. We also recorded the photon count rate for both configurations to compare against theoretical predictions. We then simulated the ideal autocorrelation for a semi-infinite homogeneous medium and added noise based on the theoretical model proposed by Zhou *et al* [4]., using parameters from our experimental settings.

For a semi-infinite medium, $g_{2}(\tau )$ can be simplified to $g_{2}(\tau )=1+\beta e^{-2\Gamma \tau }$ , where Γ is the decay rate. Theoretical noise in the measurement, expressed as the standard deviation σ(τ) is modeled as:

(7) $$\begin{array}{rl} \sigma (\tau ) & =\sqrt{\frac{T}{t}}\;[\beta^{2}\frac{(1+e^{-2\Gamma T})(1+e^{-2\Gamma \tau })+2m(1-e^{-2\Gamma T})e^{-2\Gamma \tau }}{\left(1-e^{-2\Gamma T}\right)}\\ & {+2\langle n\rangle^{-1}\beta (1+e^{-2\Gamma \tau })+\langle n\rangle^{-2}(1+\beta e^{-\Gamma \tau })]}^{\frac{1}{2}}, \end{array}$$

where *T* is the correlator bin width, determined by our time-tagger module: (SPC-QC-104, 6.145 ns for the first 16 delays, tripling every subsequent 16 values), *t* is the integration time, *m* is the bin index, ⟨n⟩ is the average photon count in the bin time ( ⟨n⟩=IT , where *I* is the detected photon count rate). We then compared the noise performance between the LLG and MMF configurations and validated the results against the theoretical noise model. Moreover, we characterized the measurement SNR, defined as SNR $=(g_{2}(\tau )-1)/\sigma (\tau )$ . Following the beam uniformity measurements in Section 2.3, the laser power levels for the LLG and MMF configurations were adjusted to yield comparable peak beam intensities to compare the SNR improvements.

### 2.5. In vivo experiments

To assess the feasibility and performance of the LLG-based DCS system in capturing physiological vascular responses, we conducted a cuff-induced ischemia experiment on a healthy adult male subject (28-year-old), following established protocols [6]. The LLG delivered an output power of 31 mW, corresponding to a 5 mm diameter illumination spot. Ethical approval for the study was granted by the Biomedical Engineering Departmental Ethics Committee at the University of Strathclyde.

The probe was securely positioned on the dorsal side of the forearm using a 3D-printed clamp and nylon straps to maintain stable contact and consistent source–detector geometry. At *ρ* = 20 mm, each measurement had an integration time of 2 s. The protocol consisted of three consecutive phases, each lasting approximately 40 s:

(1) **Baseline:** blood flow was recorded under resting conditions to establish baseline values;
(2) **Ischemia (cuff occlusion):** a pressure cuff on the upper forearm was inflated to 200 mmHg for 40 s, temporarily restricting blood flow and inducing localized ischemia;
(3) **Recovery:** The pressure was released, resulting in a transient hyperemic response where blood flow temporarily exceeded the baseline level before gradually returning to baseline conditions.

## 3. Results

### 3.1 Consistency analysis of extracted **$D_{B}$**

**Fig. 2.**
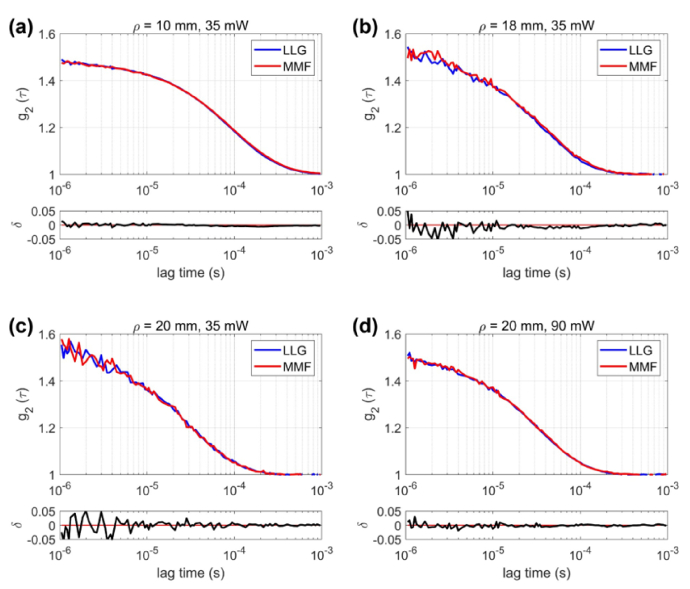
Representative autocorrelation curves g_2_(τ) obtained from LLG and MMF illumination under four conditions: (a) 10 mm, 35 mW; (b) 18 mm, 35 mW; (c) 20 mm, 35 mW; and (d) 20 mm, 90 mW. Blue and red lines correspond to LLG and MMF, respectively, and the lower panels show their residual differences (δ). The curves overlap closely, indicating consistent fitting results between the two illumination methods.

Experiments were performed at six *ρ*, and two laser power levels (35 mW and 91 mW at ρ = 20 mm) to evaluate the consistency of the LLG and MMF illumination methods in terms of $g_{2}$ curve fitting and the extracted $D_{B}$ . Under the same laser power, the measured output power of the LLG was approximately 10% lower than that of the MMF due to LLG’s lower transmission efficiency. For varying laser output power (*ρ* = 20 mm) and varying *ρ* (laser power was 35 mW), the recovered $D_{B}$ and its standard deviation were summarized in Table 1 and Table 2, respectively. In each case, the $g_{2}$ curves (Fig. 2) measured with the LLG overlap substantially with those obtained with the MMF. Only minor residuals (δ) were observed across all delay times. The Tab.2 test environment temperature was recorded as 19.6 ± 0.5°C.

**Table 1.** The measured **$D_{B}$ **, its standard deviation, and total photon count under different laser power levels.

ρ (mm)	Output Power^*a*^ (mW)	Illumination Method	Mean $D_{B}$ ( $\times 10^{-7}$ cm^2^/s)	Std. Dev. ^*b*^ ( $\times 10^{-7}$ cm^2^/s)	PhotonCounts (10 s interval, ×10^5^)
20	35	MMF	3.29	0.09	5.1
20	35	LLG	3.12	0.02	3.8
20	91	MMF	2.95	0.05	13.2
20	91	LLG	2.95	0.02	9.9

^*a*^ The output power refers to the laser output power and was tuned by applying a neutral density filter.

^*b*^ The *D_B_* value represents the mean of three measurements, with a corresponding standard deviation.

**Table 2.** The measured $D_{B}$ , its standard deviation, and total photon count at different ρ .^*a*^

ρ (mm)	Output Power (mW)	Illumination Method	Mean $D_{B}$ ( $\times 10^{-7}$ cm^2^/s)	Std. Dev. ( $\times 10^{-7}$ cm^2^/s)	PhotonCounts (10 s interval, ×10^5^)
10	35	MMF	2.87	0.03	39.0
10	35	LLG	2.75	0.03	36.2
12	35	MMF	2.85	0.02	22.6
12	35	LLG	2.62	0.03	21.6
14	35	MMF	2.75	0.04	14.8
14	35	LLG	2.76	0.04	14.3
16	35	MMF	2.85	0.02	9.2
16	35	LLG	2.73	0.04	7.5
18	35	MMF	2.86	0.07	6.9
18	35	LLG	2.89	0.04	6.1
20	35	MMF	2.81	0.07	4.6
20	35	MMF	2.80	0.08	4.3

^*a*^ Note: The *D_B_* value represents the mean of five measurements, with a corresponding standard deviation.

At short separation (ρ = 10 mm), a small difference in the fitted $D_{B}$ values between LLG and MMF was observed several times; the underlying reasons for this discrepancy are discussed in detail in the Discussion section.

**Fig. 3.**
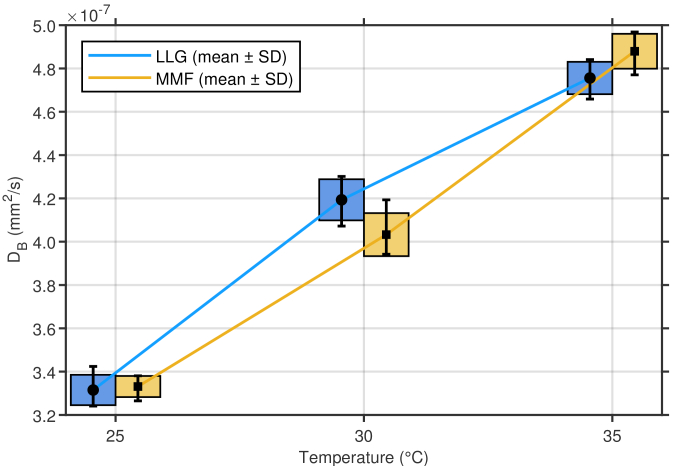
Mean $D_{B}$ values at different measurement temperatures (25, 30, and 35 °C). Rectangles denote the mean ± SD range, while the capped bars indicate the full data range (min–max). Both LLG and MMF configurations exhibit consistent increases of $D_{B}$ with temperature, confirming their equivalent sensitivity to Brownian-motion dynamics.

As shown in Fig. 3, both LLG and MMF captured the expected increase of $D_{B}$ with temperature, consistent with the Einstein–Stokes relation, with nearly identical changes across the two illumination methods.

### 3.2. Beam uniformity analysis

**Fig. 4.**
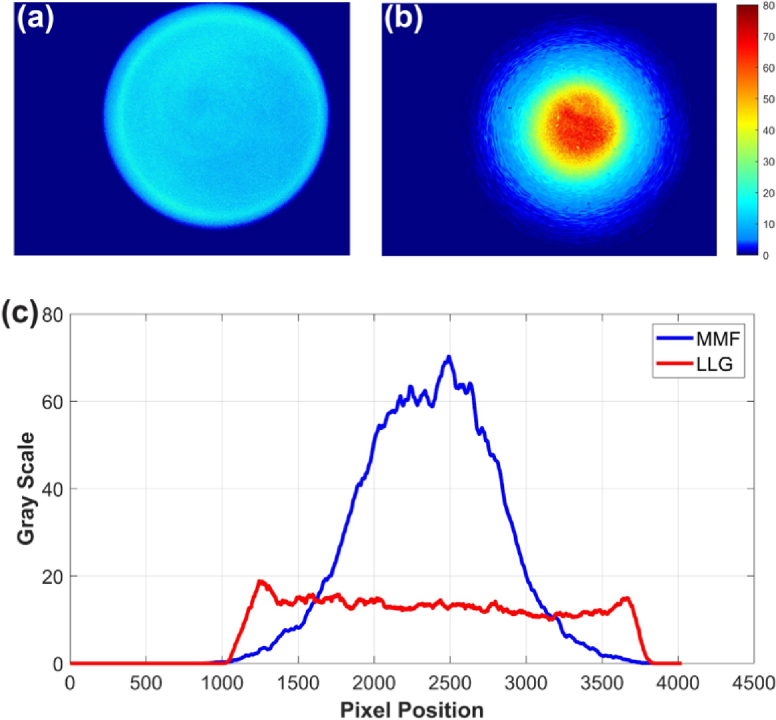
Beam profiles for (a) LLG and (b) MMF (600 µm core, NA = 0.22) illumination. (c) Intensity cross-sections along the beam center after moving-average filtering (50 pixels). The LLG shows a more uniform, flat-top distribution, whereas the MMF output retains a Gaussian-like profile.

To assess beam uniformity, we utilized a monochrome CMOS camera (Alvium 1800 U-1240M, 4024 × 3036 pixels, 1.85 × 1.85 µm pixel size) to image the beam profiles of the LLG and MMFs. An attenuator was employed to reduce the laser output power from 100 to 1 mW, ensuring consistent, unsaturated image acquisition. The total recorded 200 frames were averaged and background noise was subtracted to obtain clean, representative beam profiles, as illustrated in Fig. 4.

Additional beam profiles (200 µm, NA 0.39 and 1000 µm, NA 0.50) are shown in Supplement 1 Fig. S1. To further assess spatial uniformity, Fig. S2 (see Supplement 1) presents a quantitative comparison based on radial intensity averaging and centroid alignment.

The peak intensity for the MMF was approximately 4.2 times higher than that of the LLG under the same laser power, reflecting a substantial difference in beam uniformity. However, this does not directly imply that the total power can be increased fourfold under safety standards. However, a more uniform beam with a spot size larger than 3.5 mm can significantly increase the allowable power without exceeding the MPE limits. This distinction is relevant for safety margin and user comfort. A detailed discussion is provided in Section 4.

### 3.3. SNR analysis

**Fig. 5.**
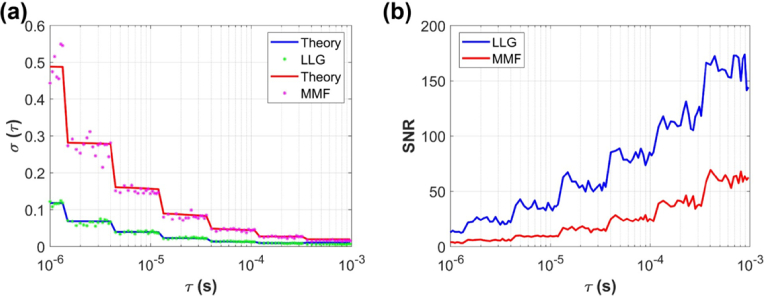
(a) Measured noise (scatter points: σ of *g_2_* as a function of *τ*) compared to the theoretical noise model (solid lines) for the LLG and MMF configurations. (b) The SNR for the LLG and MMF configurations (under the matched peak-intensity condition). The LLG configuration shows higher SNR, consistent with its increased photon counts.

As mentioned in Section 3.2, we adjusted the LLG’s output to be ∼ × 4.2 MMF’s (∼31 mW vs ∼7 mW) to match their peak power densities. Under this condition, the LLG delivered approximately 3.89 times more photon counts than the MMF (photon count for LLG and MMF are 4.48 × 10^5^, 1.15× 10^5^, respectively). To assess how this difference translated into signal quality, we computed the point-wise ratio of SNR (SNR_LLG_/SNR_MMF_) over the first 70 delay points (corresponding to *τ* up to 100 µs). The resulting average ratio was 3.68, closely matching the photon count ratio and confirming that the SNR improvement is primarily attributable to increased photon throughput, as shown in Fig. 5.

### 3.4. In vivo validation with cuff occlusion

**Fig. 6.**
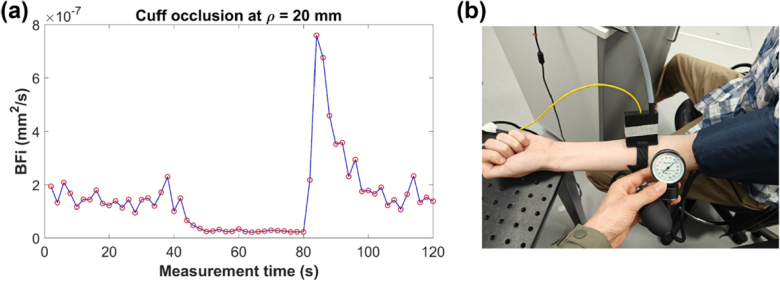
(a) Time course of the BFi during the cuff-occlusion experiment at ρ = 20 mm, showing baseline, ischemia, and recovery phases. Blood flow decreases during occlusion and exhibits a transient hyperemic overshoot after release. (b) Photograph of the portable LLG-based DCS probe attached to the subject’s forearm during measurement.

Representative results from the cuff-induced ischemia experiment using the portable LLG-based DCS system are shown in Fig. 6. The BFi time course indicates that the LLG configuration can effectively capture the physiological transitions across the baseline, ischemic, and recovery phases. The results are consistent with the expected physiological response to ischemia and reperfusion (a marked reduction in flow during occlusion followed by a hyperemic overshoot upon release) [6]. Notably, the findings show that the LLG-based DCS system is sensitive to dynamic vascular changes and is suitable for *in vivo* blood flow monitoring. The results further support the adoption of an LLG illumination for clinical or portable DCS applications, leveraging its higher photon count rate for improved measurement accuracy.

## 4. Discussion

*Finite-source effects at short source–detector separations*: The Green’s function solution of the correlation diffusion equation assumes a point source, whereas in our experiments the illumination area is finite. The MMF output can be approximated by a localized spot, while the LLG delivers a broader, more uniform disk distribution. This distinction is especially important at short ρ , where Table 1 shows that at ρ=10 mm the fitted $D_{B}$ values deviate, but the difference diminishes at larger separations. The CDE solution is linear in the source term, so the extended-source case can be obtained by integrating the point-source solution over the illumination area:

(8) $$G_{1}^{\text{disk}}(\rho ,\tau )=\frac{1}{\pi a^{2}}\int_{|{\text{r}}_{s}|\leq a}\;G_{1}^{\text{point}}(|\rho -{\text{r}}_{s}|,\tau )d^{2}{\text{r}}_{s},$$

Numerical evaluation shows that at ρ=10 mm the disk source produces a slower decay of $g_{2}(\tau )$ , leading to a smaller fitted $D_{B}$ . The fitted values were $2.87\times 10^{-7}\;cm^{2}/s$ (point source) versus $2.68\times 10^{-7}\;cm^{2}/s$ (disk), consistent with experiment (Fig. S4).

At larger separations the difference quickly diminishes. The dependence of this bias on separation was examined both numerically and experimentally. Supplement 1 Fig. S5 shows the simulated deviations between point- and disk-source models. Beyond 15 mm the bias decreases rapidly. Fig. S4 shows the same pattern: a clear discrepancy at 10 mm but negligible differences at longer separations.

Monte Carlo simulations using the open-source Monte Carlo eXtreme (MCX) simulation toolbox in MATLAB with a predefined diffusion coefficient further reproduced this behavior [17,14]. At ρ=10 mm, the disk source underestimated $D_{B}$ relative to the ground truth, while at ρ=20 mm the fitted results converged (see Fig. S6). Other experimental factors such as phantom temperature fluctuations, ambient light leakage, limitations of the semi-infinite model at very short separations, phantom inhomogeneity, and background motion noise may also contribute to variability. Nonetheless, the combined experimental and simulation results consistently indicate that finite-source effects are the dominant cause of the observed short-separation bias.

*Beam profile and illumination characteristics*: Beam uniformity was assessed using a CMOS camera. Although a neutral density filter was applied to prevent saturation, local overexposure near the beam center may still have affected the measurement accuracy. Future work should consider using a dedicated laser beam profiler for more precise characterization. During measurement, we kept the distal end of the LLG relatively straight; however, bending the LLG may affect the beam shape and uniformity. This potential influence deserves further investigation, such as through simulations or controlled experiments under various bending conditions, to better understand its impact on system performance.

*MPE and Beam Profiles:* According to ANSI Z136.1 (Section 9.2.2.1 and Section 8) [18] and IEC TR 60825-14 (Section 5.3) [19], the MPE is defined based on power or energy density averaged over a fixed limiting aperture (typically 3.5 mm for skin exposure), rather than the actual beam size. When the beam diameter is smaller than 3.5 mm, the total power is averaged over the entire aperture area regardless of the spot size. Conversely, when the beam exceeds 3.5 mm, MPE is determined by the highest irradiance within any 3.5 mm sub-aperture region, typically at the beam center.

MMF illumination often results in non-uniform, Gaussian-like beam profiles. As shown in Fig. 3 and Fig. S2, for a 5 mm Gaussian beam produced by a 600 µm, NA 0.22 MMF, approximately 91.1% of the total intensity is concentrated within the central 3.5 mm region, which only accounts for half of the beam area. Consequently, increasing beam size does not substantially reduce MPE-limiting irradiance with MMFs.

In contrast, LLGs typically yield more uniform, flat-top beam profiles. For the same 5 mm beam diameter, an LLG distributes energy more evenly, thereby reducing peak irradiance. Although photon counts from the LLG were ∼20% lower compared to MMF at equal power, its uniform profile allows approximately twice the total power within MPE limits. In contrast, the MMF with 91.1% of energy confined to the center which permits only ∼1.10× total power increase. Thus, switching to LLG illumination can yield a theoretical ∼1.75× improvement in SNR.

Although peak irradiance may correlate more closely with user perception, it is not used for compliance with MPE. As noted in ANSI Z136.3 (Section 8) [20]: “While the MPEs are below known hazardous levels, eye or skin exposures at these levels may cause discomfort.” Similar effects have been reported [21]. Therefore, expanding and flattening the beam remains valuable in DCS, improving comfort and tolerance even below regulatory thresholds.

We also performed supplementary in vivo measurements using the ATLAS SPAD array to directly compare MMF as point source and LLG under MPE-constrained powers for cerebral blood flow measurement at ρ=25 mm [22]. The detailed introduction of the SPAD-DCS system can be found in our previous publication [23,24]. According to IEC standards, the maximum permissible exposure is 28 mW for a point source MMF and 57 mW for a 5 mm diameter flat-top beam such as that delivered by the LLG. In our tests, the output powers were set to 19 mW (MMF) and 42 mW (LLG), both below their respective thresholds. These results, presented in Supplement 1 Fig. S3, demonstrate that LLG illumination yields clearer pulsatile signals than MMF, consistent with the expected ∼2.05× difference in permissible power between a point source and a 5 mm beam.

*System robustness and practical constraints*: All tests in this study were performed under stable, motion-free laboratory conditions; under these conditions, no adverse effects from the LLG’s internal liquid medium were observed. Nevertheless, the robustness of the LLG-based system under scenarios involving motion or vibration remains uncertain and should be evaluated in future work. Furthermore, practical mechanical constraints must be considered: the LLG5-4Z has a relatively large diameter and limited bending flexibility (minimum bend radius ∼60 mm), which is significantly greater than that of a typical MMF. For portable or wearable DCS implementations, these factors present handling challenges. Lastly, our measurements were limited to relatively small ρ (≤ 20 mm) due to the modest detection efficiency of the detector used. Future studies should explore a larger ρ to assess blood flow in deep tissues.

*Future directions*: First, identifying cost-effective alternatives to LLGs (currently ∼£400 per unit and thus more expensive than MMFs at ∼£100) would improve the accessibility of this system. In practice, options such as rigid light guides, fiber bundles, or MMFs with microlens surfaces may offer lower transmission efficiency but at much lower cost, representing promising directions for future development. Second, further optimization of the beam quality, either through improved illumination design or the integration of additional optical elements, could yield even more uniform beam spots and a higher SNR. Third, since our current validation experiments were limited to a semi-infinite homogeneous medium, further work is needed to determine whether similar performance can be maintained in more complex tissue environments. This could be explored via Monte Carlo simulations and experiments using multi-layer phantoms with well-defined optical properties.

Lastly, in this study we only explored the feasibility of using LLGs for illumination. In diffuse speckle contrast analysis (DSCA), several recent reports have demonstrated that combining multi-mode fibers with CMOS detectors can achieve higher-SNR BFi extraction at lower cost compared to conventional single-mode detection [25,26]. Given that the cross-sectional area of an LLG is tens of times larger than that of an MMF, it has the potential to substantially improve system SNR in future implementations. Recent advances in DCS and related dynamic light scattering techniques suggest promising directions for extending the potential of our liquid light guide approach [27]. Zherebtsov *et al.* (2024) introduced entropy-based analysis and PCA to transcranial blood flow imaging, achieving sensitivity comparable to traditional DCS while enabling the detection of subtle hemodynamic changes induced by pharmacological interventions [28]. Moreover, their work on interferometric speckle visibility spectroscopy (ISVS) highlights how interferometric detection can overcome low-photon-count limitations and allow high-SNR blood flow measurements under low illumination conditions. Integrating such advanced analysis and detection strategies with the liquid light guide illumination proposed here could further improve the SNR and penetration depth of cerebral blood flow measurements, especially under the same safety exposure limits.

## 5. Conclusion

This study introduced a novel DCS system utilizing an LLG as an alternative illumination method to MMFs. By generating a more uniform beam profile, the LLG-based approach enables a significant increase in photon count rate within the safe exposure limits for human skin. We first validated measurement consistency between LLG and MMF setups using phantom experiments. We then assessed the beam uniformity provided by the LLG relative to the MMF (at matched beam size). Furthermore, phantom measurements under matched peak intensity conditions revealed that the LLG-based DCS system offers improved photon throughput and a consequent enhancement in SNR, and theoretical calculation of an ∼1.75-fold SNR enhancement under equivalent MPE. Finally, the performance and effectiveness of the LLG-based system were validated in a cuff-induced ischemia experiment on a human forearm, demonstrating its ability to capture the expected physiological blood flow responses. The improvement in SNR not only supports more robust measurements under standard conditions, but also opens up possibilities for extending ρ and improving the accuracy of blood flow quantification in deeper tissues.

## Supplemental information

Supplement 1 Supplementary materials https://doi.org/10.6084/m9.figshare.30452864

## Data Availability

Data underlying the results presented in this paper are not publicly available at this time but may be obtained from the authors upon reasonable request.

## Supplemental document

See Supplement 1 for supporting content.
