# Supplementary material for: Assessing liquid light guides in diffuse correlation spectroscopy systems
Source: Biomed Opt Express. 2025 Nov 4;16(12):4957–69. doi: 10.1364/BOE.571835 (PMC12698105; doi:10.1364/BOE.571835)
Supplement: Supplementary file 1 [file boe-16-12-4957-s001.pdf]

Supplemental Document

Biomedical Optics EXPRESS

Assessing liquid light guides in diffuse correlation spectroscopy systems: supplement

YUANZHE ZHANG,[1,†] MINGLIANG PAN,[1,†] CHENXU LI,[1] ZIAO JIAO,[1] YUANYUAN HUA,[2] AHMET T. ERDOGAN,[2] ROBERT K. HENDERSON,[2] AND DAVID DAY-UEI LI[1,*]

[1] *University of Strathclyde, Department of Biomedical Engineering, Glasgow, UK*
[2] *University of Edinburgh, Institute for Micro and Nano Systems (IMNS), School of Engineering, Edinburgh, UK*
[†] *These authors contributed equally to this work and are considered co-first authors.*
[*] *david.li@strath.ac.uk*

This supplement published with Optica Publishing Group on 4 November 2025 by The Authors under the terms of the Creative Commons Attribution 4.0 License in the format provided by the authors and unedited. Further distribution of this work must maintain attribution to the author(s) and the published article's title, journal citation, and DOI.

Supplement DOI: https://doi.org/10.6084/m9.figshare.30452864

Parent Article DOI: https://doi.org/10.1364/BOE.571835

Supplementary Materials for
" Assessing liquid light guides in diffuse correlation spectroscopy systems"

Yuanzhe Zhang[1†], Mingliang Pan[1†], Chenxu Li[1], Ziao Jiao[1], Yuanyuan Hua[2], Ahmet T. Erdogan[2], Robert K. Henderson[2], and David Day-Uei Li[1*]

[1]*University of Strathclyde, Department of Biomedical Engineering, Glasgow, UK*

[2]*University of Edinburgh, Institute for Micro and Nano Systems (IMNS), School of Engineering, Edinburgh, UK*

† These authors contributed equally to this work and are considered co-first authors.

* *david.li@strath.ac.uk*

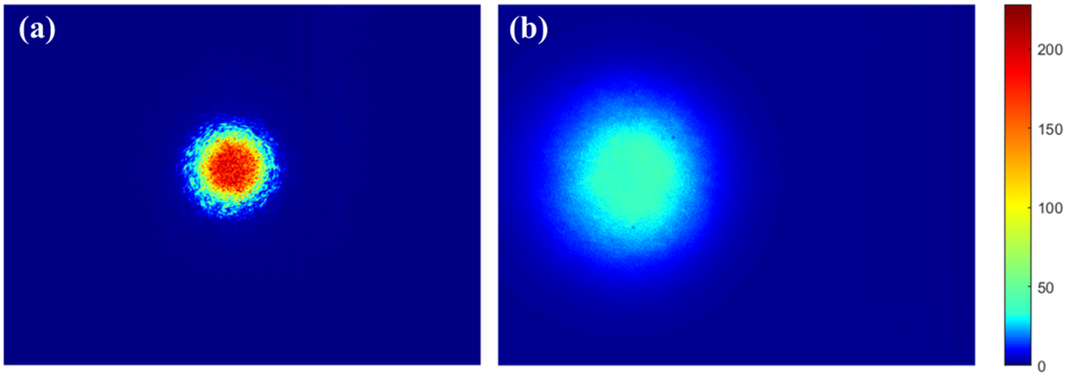

Supplementary Figure S1. The beam profiles of (a) 200 µm (NA=0.39) and (b) 1000 µm (NA=0.50) multimode fibers. The beam profiles were recorded after passing through an neutral density filter, adjusted to produce similar beam diameters at the detection plane. (a) The 200 µm fiber output shows a highly concentrated intensity distribution with near-saturation at the center. (b) The 1000 µm fiber output displays a more uniform spatial distribution across the beam spot. Both profiles exhibit a characteristic central high-intensity region that gradually decreases toward the edges.

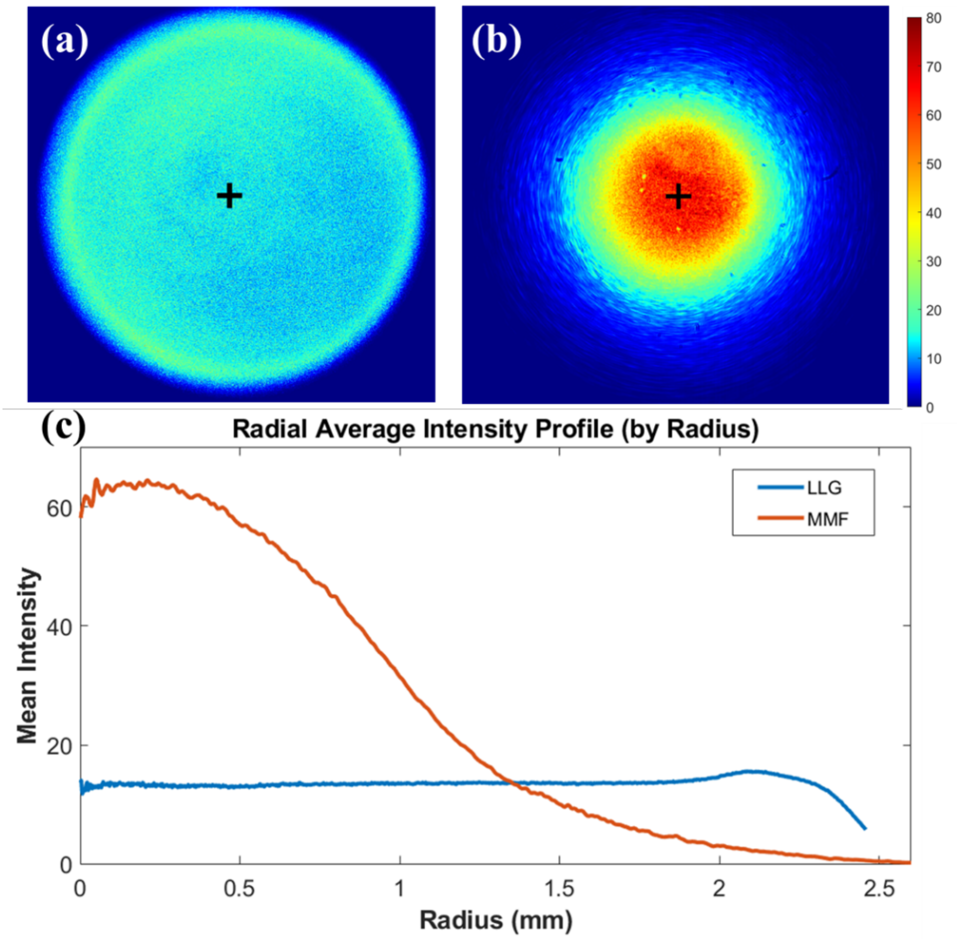

Supplementary Figure S2. The beam profiles and corresponding radial intensity distributions for LLG and MMF configurations. (a) The beam profile recorded from the LLG illumination. (b) The beam profile recorded from the MMF illumination. The center of the beam (marked by a black cross) was determined using an intensity-weighted centroid algorithm. (c) Radial average intensity profiles extracted from (a) and (b), showing the mean intensity as a function of the radial distance. The MMF profile exhibits a high peak intensity near the center and a sharp decay, whereas the LLG distribution is more uniform across the beam cross-section. The maximum intensity observed for the MMF was 64.6, compared to 15.4 for the LLG.

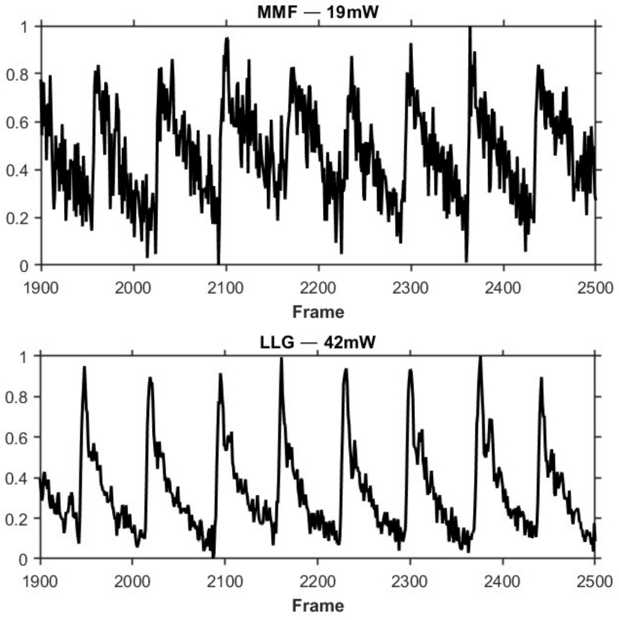

Supplementary Figure S3. Normalized recovered cerebral blood flow waveform measured in vivo on the forehead using the ATLAS SPAD array with (a) MMF point-source illumination at 19 mW (MPE threshold 28 mW) and (b) LLG 5 mm flat-top illumination at 42 mW (MPE threshold 57 mW). The decay rate was quantified within 39.68 μs by subtracting the last point from the first point of the autocorrelation curve. Both conditions complied with IEC safety limits. The LLG case shows improved signal clarity, consistent with its larger permissible power under MPE rules.

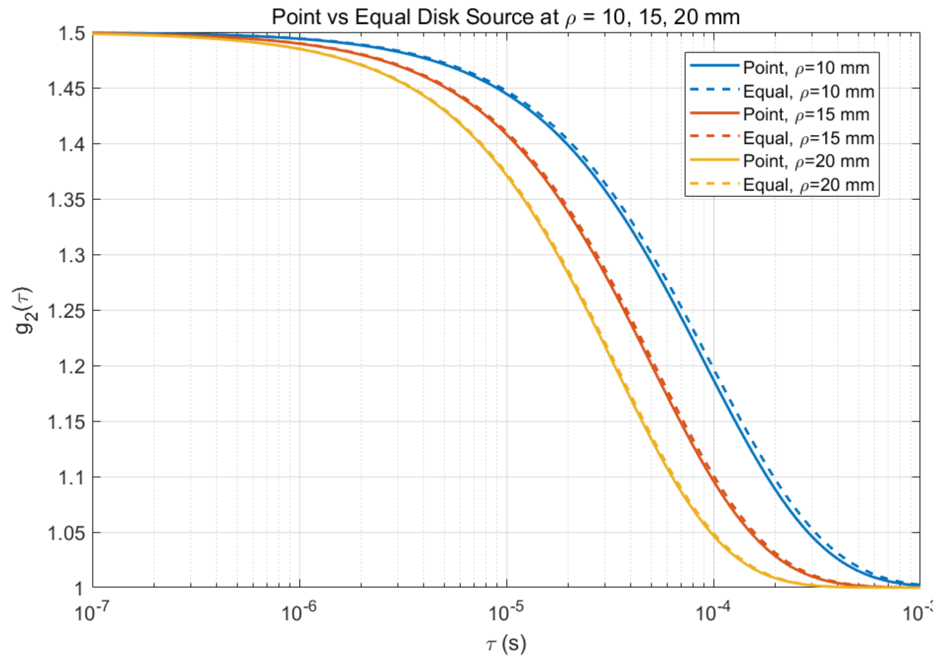

Supplementary Figure S4. Simulated autocorrelation functions g_2 for point-source and disk (top-hat) illumination at source–detector separations of ρ = 10, 15, and 20 mm. At ρ = 10 mm, the disk source shows a slower decay of g_2, which leads to a smaller fitted diffusion coefficient D_B. At larger separations ($\rho \geq 15$ mm), the difference between point and disk sources diminishes, indicating that finite-source effects are most significant at short separations.

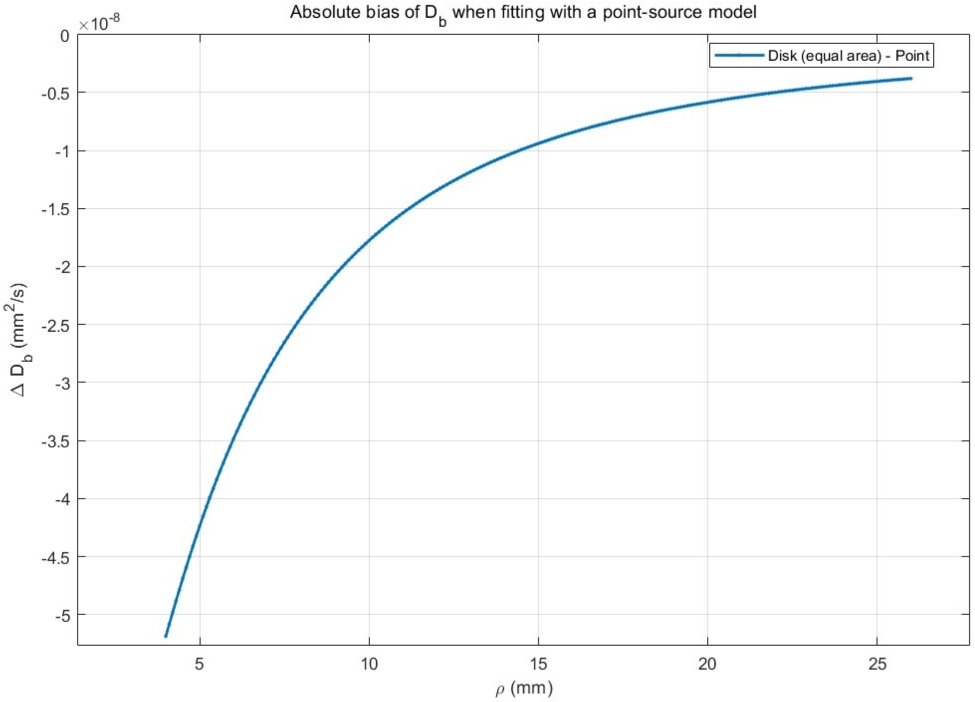

Supplementary Figure S5. Absolute bias of the fitted diffusion coefficient D_B when applying a point-source model to disk-source data, shown as a function of source–detector separation ρ. The bias is most pronounced at short separations, with $D_B \approx -5\times10^{-8}$ mm²/s at ρ ≈ 5 mm, and gradually decreases in magnitude as ρ increases, approaching zero by ρ ≈ 25 mm. This confirms that finite-source effects primarily influence short-separation measurements and become negligible at longer distances.

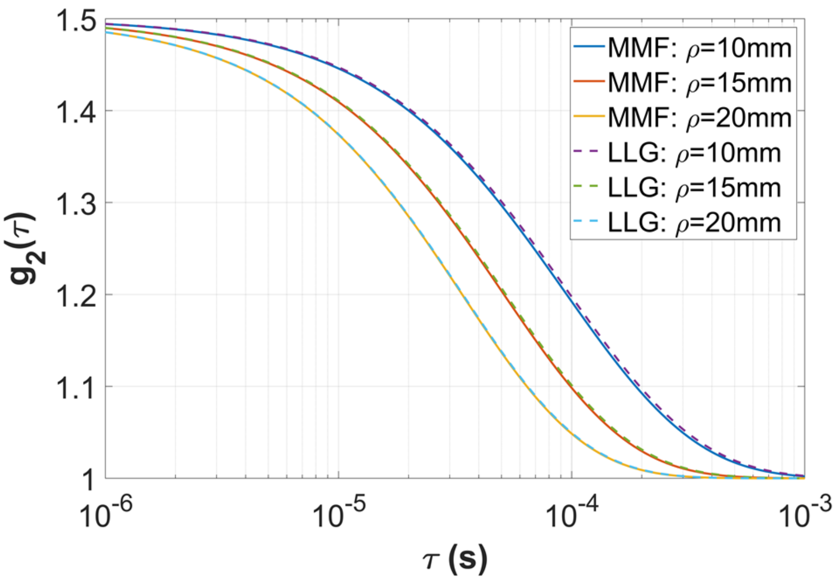

Supplementary Figure S6. To compare the influence of different source types on the ACF patterns, we simulated a point source (0.16 rad, corresponding to 0.22 NA) for the MMF and a disk source (radius = 2.5 mm) for the LLG. The simulations were performed using the Monte Carlo eXtreme (MCX) toolbox in MATLAB, with 10e9 photons at ρ of 10, 15, and 20 mm, DB is set to $2.87 \times 10^{-7}\text{cm}^2/\text{s}$. The recorded photon transport and pathlength data were then used to calculate the light field ACF. At $\rho = 10$ mm, the fitted values were $2.77 \times 10^{-7}\text{cm}^2/\text{s}$ for

MMF and $2.67 \times 10^{-7} \mathrm{cm}^2/\mathrm{s}$ for LLG, showing a clear underestimation relative to the ground truth. At $\rho = 20$ mm, the fitted results ($2.81 \times 10^{-7}\ \mathrm{cm}^2/\mathrm{s}$ vs. $2.79 \times 10^{-7} \mathrm{cm}^2/\mathrm{s}$) converged and closely matched the input value.
